# Preclinical modeling of lower-grade gliomas

**DOI:** 10.3389/fonc.2023.1139383

**Published:** 2023-03-27

**Authors:** Lilly W. Tang, Arka N. Mallela, Hansen Deng, Timothy E. Richardson, Shawn L. Hervey-Jumper, Samuel K. McBrayer, Kalil G. Abdullah

**Affiliations:** ^1^ Physician Scientist Training Program, University of Pittsburgh School of Medicine, Pittsburgh, PA, United States; ^2^ Department of Neurosurgery, University of Pittsburgh Medical Center, Pittsburgh, PA, United States; ^3^ Hillman Cancer Center, University of Pittsburgh Medical Center, Pittsburgh, PA, United States; ^4^ Department of Pathology, Cell and Molecular Based Medicine, Icahn School of Medicine at Mount Sinai, New York, NY, United States; ^5^ Department of Neurological Surgery, University of California San Francisco, San Francisco, CA, United States; ^6^ Children's Medical Center Research Institute, University of Texas Southwestern Medical Center, Dallas, TX, United States

**Keywords:** glioma, organoids, preclinical models, lower-grade glioma, cerebral organoids, patient-derived 3D tumor explants

## Abstract

Models for human gliomas prove critical not only to advancing our understanding of glioma biology but also to facilitate the development of therapeutic modalities. Specifically, creating lower-grade glioma (LGG) models has been challenging, contributing to few investigations and the minimal progress in standard treatment over the past decade. In order to reliably predict and validate the efficacies of novel treatments, however, LGG models need to adhere to specific standards that recapitulate tumor genetic aberrations and micro-environment. This underscores the need to revisit existing models of LGG and explore prospective models that may bridge the gap between preclinical insights and clinical translation. This review first outlines a set of criteria aimed to address the current challenges hindering model development. We then evaluate the strengths and weaknesses of existing preclinical models of LGG with respect to these established standards. To conclude, the review discusses potential future directions for integrating existing models to maximize the exploration of disease mechanisms and therapeutics development.

## 1 Introduction

Lower-grade gliomas (LGG), classified by WHO as grade 2/3, are infiltrative, primary brain tumors that are nearly uniformly fatal. Unlike in patients that present with grade 4 gliomas, glioblastomas (GBMs), patients with LGG have a variable and extended time horizon from diagnosis and treatment until tumor progression. This relatively favorable prognosis creates challenges for LGG management because treatment options must be carefully considered for patients who may spend a decade or more without substantial clinical symptoms after upfront resection or treatment. As a result, current evaluation of efficacy relies heavily on either treatment at the time of diagnosis or long-term health outcome measurements. Robust, faithful preclinical LGG models are needed to facilitate testing of therapeutic strategies and circumvent barriers to determining treatment response, which are critical for improving quality of life and survival outcomes for patients with LGG.

There is a paucity of LGG models that support preclinical target identification and drug screening studies. Given that the standard treatment for LGG has remained the same for the past 15 years (1), difficulty in modeling these tumors impedes development of novel therapies. This situation underscores the need to leverage existing models of LGG and explore new approaches that may bridge the gap between preclinical insights and clinical translation. Current LGG models include established and patient derived cell lines, murine models, and 3D glioma organoid cultures. Each model has unique strengths and limitations that must be taken into account when selecting models for laboratory-based experimentation. In this review, we highlight recent advances in LGG modeling capability, evaluate existing preclinical models of LGG, and discuss future directions.

## 2 Genetic profile

Preclinical brain tumor models play a crucial role in therapeutic development because of their usefulness in predicting patient response and identifying biomarkers of treatment efficacy. Since up to 80% of LGGs harbor mutations in genes encoding isocitrate dehydrogenase enzymes *(IDH1/IDH2) (* 2), preserving these driver mutations in model systems is crucial. The co-occurrence of IDH mutations with other genetic aberrations such as 1p/19q co-deletion, loss of function mutations in the alpha thalassemia X-linked intellectual disability gene (*ATR-X*), and inactivation of the tumor suppressor protein 53 (TP53) serve as distinct markers enabling genetic and histopathological classification of LGG tumors into oligodendrogliomas and astrocytomas (3). As such, incorporating clinically relevant combinations of genomic alterations in LGG models is necessary to accurately portray specific subtypes of glioma (4).

In IDH wild-type cells, IDH enzymes convert isocitrate into α-ketoglutarate (α-KG). In cells with IDH mutations, mutant IDH enzymes lose wild-type activity and instead catalyze an alternative reaction: converting α-KG to the *R* enantiomer of 2-hydroxyglutarate [(*R*)-2HG] (5). Consequently, (*R*)-2HG accumulation reprograms epigenetic, metabolic, and immunological pathways. These effects collectively contribute to the phenotypes observed in IDH-mutant LGGs. Therefore, it is desirable for models of LGG to recapitulate not only the IDH1/2 mutations but also the production of the oncometabolite (*R*)-2HG in order to accurately reflect LGG tumor biology.

## 3 Tumor microenvironment supports hypoxia

Glioma cells share a close relationship with the surrounding tumor microenvironment (TME), consisting of blood and lymphatic vasculature, glia, neurons, immune cells, and structural components of the extracellular matrix (6). Like most solid cancers, LGGs display features of microenvironmental remodeling that promote tumor progression.

Tumors growing more rapidly than surrounding tissue frequently have restricted access to oxygen. These hypoxic conditions can trigger compensatory responses, particularly the stabilization of hypoxia-inducible factors 1α and 2α (HIF1α and HIF2α). HIF1α induces upregulation of proangiogenic genes such as platelet-derived growth factor (PDGF), which activates downstream oncogenic pathways of phosphatidylinositol 3-kinase/protein kinase B (PI3K/AKT) and mitogen-activated protein kinase/extracellular signal-regulated kinase (MAPK/RAS) signaling (7). However, HIF1α also exerts tumor suppressive effects in glial cells (8), and (*R*)-2HG has been shown to stimulate HIF1α degradation as part of the oncogenic program associated with IDH mutations in glioma (9, 10). More specifically, IDH mutant gliomas display under-expression of HIF1α-responsive genes, many of which that encode enzymes essential for glycolysis (*SLC2A1, PDK1, LDHA*) (11). These downregulations suggest a limited glycolytic capacity unique to IDH mutant gliomas that may explain their slow progression. In contrast, HIF2α appears to play a clearer oncogenic role in glioma. HIF2α is stabilized in GSCs at moderate levels of hypoxia, whereas HIF1α protein accumulation is only observed under more severe oxygen deprivation (12). HIF2α stabilization is sufficient to enhance glioma cell tumorigenicity, which appears to be partly attributable to its ability to upregulate stemness-related genes (13). Setting aside the specific role of hypoxia in triggering changes in HIF1α and/or HIF2α expression, hypoxia has also been reported to selectively upregulate programmed death-ligand 1 (PD-L1) on myeloid-derived suppressor cells (MDSCs) and macrophages in the TME, allowing glioma cells to escape immune checkpoints (14). Together, the capacity to recapitulate hypoxic conditions in LGG models is an important consideration given its impact on the angiogenic, immunosuppressive, and acidic nature of the tumor microenvironment.

## 4 LGG cell lines

Cell lines are a cornerstone of preclinical cancer research. Adherent cell lines, commonly grown in fetal bovine serum-containing medium, are cost-effective, easily manipulable, and can be used for high-throughput experimentation (15). However, these cell lines frequently undergo changes in differentiation status during exposure to serum, rendering them poorly suited for studies related to cell state changes or stemness-related phenotypes in cancer. One adherent, serum-cultured LGG line that has been commonly used is the HOG glioma line, which was established from a human oligodendroglioma specimen two decades ago by Post and Dawson (16). Although this cell line has proved useful in supporting high-throughput studies, it is IDH wild-type (thus contrasting expectations for an oligodendroglioma-derived line) and lacks expression of stemness-related genes commonly observed in glioma stem-like cells (GSCs) such as CD133, nestin, and Olig2 (17). Recently, Yuan and colleagues reported a successful method for producing adherent cell cultures from LGG tumor samples involving conditional reprogramming. This method employed fibroblast-conditioned media supplemented with a Rho kinase inhibitor. This study focused on creating LGG cell lines from pediatric tumor specimens that lacked *IDH* mutations (18). As such, it remains to be determined whether the conditional reprogramming approach could also be successfully applied to create LGG cell lines from IDH mutant adult glioma tissue samples.

Another class of cell lines used to model LGG *in vitro* include patient-derived GSCs. These cell lines are commonly grown as suspension cultures of neurospheres in serum-free, growth factor supplemented defined media. The process of creating patient-derived GSC lines, however, is notably inefficient. Success rates for generating GSC lines from HGG patient samples range between 5-10%, and GSC lines produced from IDH mutant LGG specimens are highly uncommon (19). As a result, producing LGG GSC lines that retain stemness profiles and common genomic alterations has been a major challenge in the neuro-oncology research field.

Recently, Kelly and colleagues successfully established two GSC lines from adult LGG oligodendroglioma tissues, BT054 and BT088, that preserved pathognomonic co-deletion of 1p/19q chromosomal arms (20). However, only one cell line, BT054, displayed an IDH1 mutation at codon R132. Meanwhile, implanting BT054 cells into immunocompromised mice has not established reliable xenografts, limiting the utility of this model for *in vivo* studies of glioma biology (20). Notably, Rohle and colleagues generated an oligodendroglioma GSC line, TS603, that displays 1p/19q loss, harbors an endogenous, heterozygous IDH1^R132H^ mutation, and reliably forms subcutaneous and intracranial xenografts (21, 22). Therefore, this constitutes a powerful model for studying LGG biology both *in vitro* and *in vivo*. Additionally, several studies have reported the success of using other IDH-mutated glioma cell lines (U87-MG-R132H, GB10, GBM164) for *in vitro* investigations (23–26). However, since the parental cells are commonly derived from GBM or other malignant tumors, which harbor genetic abnormalities rarely detected in LGG, the application of using these cell lines to investigate IDH1-mediated oncogenesis may be confounding.

One of the main drawbacks of GSC and adherent LGG cell line models is their inability to recapitulate the tumor microenvironment. For instance, nutrient conditions and cytokine/growth factor composition of these cultures deviate from the chemo-physiological conditions of the original tumor environment (20). Additionally, the diverse cell types (immune cells, pericytes, etc.) and ECM components that characterize the tumor microenvironment are not present in cell line cultures. Even with recent advances in incubation techniques (27), culturing cells under hypoxic conditions remains a complicated and time-intensive endeavor. As a result, reduced complexity and heterogeneity of LGG cell cultures are inherent limitations of these experimental model systems.

## 5 Mouse models for LGG

*In vivo* cancer modeling confers numerous advantages as an experimental tool that may provide new insights into the genetic landscape and molecular mechanisms of LGG. Since the mouse genome shares over 80% of orthologs with humans, most cancer researchers have adopted the mouse as the dominant model organism (28).

### 5.1 Xenograft models

The xenograft model has been widely used due to its relatively low costs and medium throughput advantages. Xenograft models can be further classified into two classes: cell-line xenograft (CLX) and patient-derived xenograft (PDX). Both models entail the xenotransplantation of either established cell lines or patient-derived biopsy tissue into a specified location in the mouse. However, to minimize the risks of rejection during engraftment, xenograft models utilize immune-deficient mice (29). The loss of the immune microenvironment is a significant limitation to understanding the interplay of tumor interactions with native cell types and modulations of the immune system.

Humanized mouse models may offer insights to address this challenge. Humanized mice are created by injecting human CD34+ hematopoietic stem cells into immune-deficient NOD/SCID mice (30). These mice engrafted with a humanized immune system then undergo xenotransplantation of tumor cells to become CLX/PDX models. This approach, though cost- and time-intensive, overcomes the restraints of xenograft models in research to further our understanding of immune modulation on tumorigenesis and progression.

Historically, xenograft models have been predominantly created from patient-derived cell lines to model GBM (31). In 2012, Luchman et al. successfully established one of the first IDH mutant LGG CLX models by engrafting a glioma brain tumor stem cell line (BT142) into NOD SCID mice (32). During tumor engraftment, BT142 cells maintained an endogenous, heterozygous *IDH1* mutation and I-2HG production. Unfortunately, the BT142 line was reported to have lost the endogenous *IDH1* wild-type allele during subsequent *in vitro* propagation (33). Loss of the wild-type allele has been shown to restrict mutant IDH1 activity because wild-type and mutant IDH1 enzymes form a heterodimer to driI(*R)*-2HG synthesis (34, 35). Consequently, BT142 cells lacking the *IDH1* wild-type allele display rIced(*R*)-2HG content. Since the development of the BT142 model, a few additional PDX models have successfully cultivated gliomas that not only harbor *IDH1* mutations and loss of 1p/19q genes (21, 36) but also retained parental metabolic fingerprints, infiltrative growth patterns, I high (*R*)-2HG production characteristic of LGG tumors (37).

Despite these successes, limitations persist in xenograft models. Fundamental differences between human and murine biology may affect tumor behavior. Components of immune cell signaling pathways and expression of cytokines and chemokines vary between humans and mice, potentially creating differences in the TME (38). Consequently, experimental therapeutics that produced promising preclinical results in mice have often failed to generate similar effects in humans. Although using humanized mice may address these challenges (39), this approach remains limited by its cost- and time-intensive nature and difficulties in replicating the intricacies of the human immune system. Currently, PDX/CLX models cannot fully recapitulate the entire TME, and thus can only offer partial insights into the complex oncogenic processes at play in LGG.

### 5.2 Genetically engineered mice models

Genetically engineered mice models (GEMMs) can elicit tumorigenesis through inducing genetic mutations in the mouse in a time- and cell-specific manner (40), enabling investigation across various stages of tumorigenesis. Moreover, the genetic modification methods for GEMMs preserve the murine immune system. Drawbacks of GEMMs include reduced heterogeneity of murine tumors relative to human tumors, relatively long tumor development latency relative to CLX/PDX models, and requirements for *in vivo* genetic engineering approaches. Nevertheless, given the precision and fidelity of the tumor molecular profiles in GEMMs, these models constitute a useful alternative to xenograft models in preclinical cancer research.

Producing GEMMs of IDH mutant glioma has proved to be a key challenge in the neuro-oncology research field. Initial attempts to create such a GEMM were spearheaded by Sasaki and colleagues, who created an IDH1^R132H^ knock-in allele (41). Unfortunately, activation of this allele during development led to widespread hemorrhaging in the brains of genetically engineered mice, causing substantial lethality that impeded assessment of glioma development. Bardella and colleagues created a similar allele but activated expression of the allele 5-6 weeks after birth in the neural stem and progenitor cell compartment, thus circumventing the hemorrhage phenotype associated with mutant IDH expression during brain development (42). These mice went on to display expansion of neural stem and progenitor cell populations but succumbed to hydrocephalus prior to frank glioma formation.

Together, these foundational studies suggested two possible approaches that could be taken to produce a GEMM of IDH1 mutant glioma. First, activation of the mutant IDH1 allele in a small fraction of neural stem and progenitor cells after brain development could be pursued to limit the incidence of hemorrhage and hydrocephalus. Second, expressing mutant IDH1 together with commonly co-occurring mutations in human gliomas could facilitate full malignant transformation of neural stem and progenitor cells and drive gliomagenesis. These strategies were employed by Philip and colleagues to create the first mutant IDH1-driven GEMM of high-grade glioma. In this model, they used an RCAS/TVA-based *in vivo* genetic engineering approach, pioneered by Holland et al (43), to introduce *PDGFA* and mutant *IDH1* cDNAs as well as loss-of-function mutations in *Cdkn2a*, *ATR-X*, and *Pten* into neural stem and progenitor cells. Recently, our group pursued similar strategies to produce a mutant IDH1-driven GEMM of LGG (44). We combined recombinant adeno-associated virus (AAV), *in vivo* CRISPR/Cas9 engineering, and conventional transgenic mouse modeling approaches to mutant *Idh1*, *Pik3ca*, *Trp53*, and *ATR-X* in neural stem and progenitor cells in the brains of adult mice. These mice went on to develop LGGs that resemble grade 3 astrocytomas in human patients. Importantly, efficient generation of autochthonous gliomas in this GEMM required the *Idh1* mutant allele.

Alternatively, Nunez and colleagues successfully produced one of the only GEMM for LGG by using transposons-directed expression of mutant IDH1^R132H^, *Trp53* and *ATR-X* in mice (45). This sleeping-beauty glioma model showed upregulation of the ATM signaling pathway that enhanced DNA damage repair capacity and elicited tumor resistance to radiotherapy. Consequent pharmacological inhibition of the ATM pathway restored the tumor’s radiosensitivity, suggesting the translational potential to improve radiotherapy outcomes for patients with IDH1 gliomas harboring similar mutations.

Taken collectively, these recent advances in producing genetically faithful GEMMs of IDH1 mutant glioma open new avenues to explore how this oncogene promotes gliomagenesis and creates therapeutic opportunities.

## 6 Organoid models for LGG

Three-dimensional human organoids may serve as complementary models to improve precision oncology and overcome limitations of cell culture and murine models of LGG. An organoid is an organized three-dimensional structure originating from murine or patient-derived stem cells or primary tissue. The first models of physiological cerebral organoids were created from human pluripotent stem cells by Lancaster et al. (46) This approach was then extended to create patient-derived organoid models of GBM (47, 48) and, more recently, LGG tumors (15).

Traditionally, organoids have been cultured as clonal stem cells induced by cofactors to create 3D organotypic structures in a gel-like extracellular matrix. However, while these provide a closer approximation of the TME, these cells remain clonal. The recent development of patient-derived, surgically explanted organoids (SXOs) from parcellating fresh cancer tissue without significant alteration has enabled models that capture the cellular heterogeneity and cell-cell interactions observed in parental tumors. Notably, Jacob et al. successfully produced GBM SXOs from fresh tissue samples cultured in media containing only supportive supplements, insulin, and antibiotics with no additional extracellular matrix substitute or exogenous growth factors (48). These GBM SXOs retained parental histological and immunological hallmarks, hypoxia gradients, and spatial heterogeneity of neoplastic and immune cells. SXOs were xenografted into immunodeficient mice to successfully recapitulate key features of human GBM tumors

**Figure 1:**
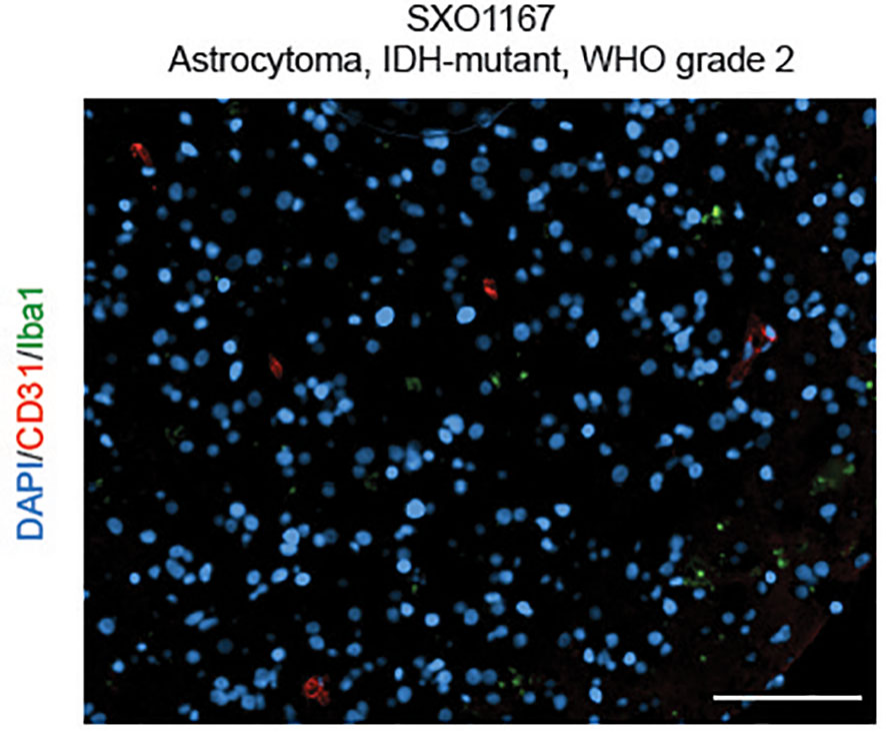
Surgically eXplanted Organoid from an astrocytoma, IDH-mutant, WHO Grade 2 (SXO1167). Organoids were cultured for 4 weeks, then cryopreserved, reanimated, and cultured for one week prior to analysis. Organoid was evaluated by CD31/Iba1 IF. Red = CD31, blue = DAPI, and green = Iba1. Scale bar = 100 µm.

Utilizing similar methodologies under modified oxygen and culture conditions, we developed a collection of SXO models of LGG tumors (15) (**Figure 1**), including organoids derived from astrocytomas (WHO grades 1-4) and oligodendrogliomas (WHO grades 2-3). These SXOs retained mutations harbored by parental tumors, including those in *IDH1, IDH2, TP53, NOTCH1, NOTCH2, CIC*, and *ATR-X* genes. They also preserved (*R*)-2HG accumulation, stemness, proliferation, and vascular content profiles of parental tumors. LGG SXOs maintained fidelity over long-term culture and throughout cryopreservation and reintroduction into culture. This methodology offers a promising new way to generate patient-derived LGG models and an effective new platform to evaluate therapeutics and investigate LGG molecular mechanisms within the context of the tumor microenvironment.

Taken together, three-dimensional organoid cultures have the ability to both reproduce tumor-specific biological and compositional properties while also allowing for high-throughput drug screening manipulation. Investigating vulnerabilities in LGG SXO models may foster identification of new molecular targets for therapeutic intervention in this disease.

## 7 Conclusion

Reliable LGG models are critical in advancing the present understanding of oncogenic molecular mechanisms, supporting preclinical therapeutic screenings, and evaluating consequent clinical treatment responses. The unique genetic profile (mutations in *IDH1, IDH2, ATR-X, 1p/19q* deletion) and complexities of the tumor microenvironment (hypoxic conditions, cell-cell/cell-stroma interactions) all contribute to the scientific challenges of constructing high fidelity LGG models.

Current preclinical models can be classified into three categories: cell lines, animal models, and 3D organoid cultures. In contrast to more widely available HGG models across each category, few models exist that accurately replicate the molecular characteristics of LGG. Namely, adherent and GSC lines, PDX/CLX models, GEMMs, and SXOs have been the primary methodologies used for preclinical testing. Considering the respective advantages and limitations of each model, integrating these preclinical models to complement each other may yield novel avenues to help understand the relationships linking glial, neuronal, and immune cells in the LGG tumor microenvironment. This integration of LGG models holds immense potential to facilitate large-scale preclinical screening for therapeutics with the goal of expediting translation of promising therapies to the bedside.

## Author contributions

The authors confirm contribution to the paper as follows: Conceptualization & design: LT, AM, KA Draft Manuscript preparation: LT Review & Approval: HD, TR, SH-J, SB. All authors contributed to the article and approved the submitted version.
